# Greigite nanocrystals produced by hyperthermophilic archaea of *Thermococcales* order

**DOI:** 10.1371/journal.pone.0201549

**Published:** 2018-08-02

**Authors:** Aurore Gorlas, Pierre Jacquemot, Jean-Michel Guigner, Sukhvinder Gill, Patrick Forterre, François Guyot

**Affiliations:** 1 Institut de Biologie Intégrative de la cellule, Laboratoire de Biologie Cellulaire des Archaea, UMR8621/CNRS, Orsay, France; 2 Institut de Minéralogie, de Physique des Matériaux et de Cosmochimie, Sorbonne Universités, Université Pierre et Marie Curie, UMR 7590 CNRS, Institut de Recherche pour le Développement, Museum National d'Histoire Naturelle, Paris, France; 3 Institut Pasteur, Laboratoire de Biologie Moléculaire du Gène chez les Extrémophiles, Paris, France; The University of Akron, UNITED STATES

## Abstract

Interactions between hyperthermophilic archaea and minerals occur in hydrothermal deep-sea vents, one of the most extreme environments for life on Earth. These interactions occur in the internal pores and at surfaces of active hydrothermal chimneys. In this study, we show that, at 85°C, *Thermococcales*, the predominant hyperthermophilic microorganisms inhabiting hot parts of hydrothermal deep-sea vents, produce greigite nanocrystals (Fe_3_S_4_) on extracellular polymeric substances, and that an amorphous iron phosphate acts as a precursor phase. Greigite, although a minor component of chimneys, is a recognized catalyst for CO_2_ reduction thus implying that *Thermococcales* may influence the balance of CO_2_ in hydrothermal ecosystems. We propose that observation of greigite nanocrystals on extracellular polymeric substances could provide a signature of hyperthermophilic life in hydrothermal deep-sea vents.

## Introduction

Greigite (Fe_3_S_4_) is an iron sulfide mineral [1] present in various terrestrial natural environments, such as sediments or hydrothermal deep-sea vents [2, 3]. In vitro experiments mimicking hydrothermal systems have shown that greigite is an intermediate in both the polysulfide (zero valent sulfur) and sulfide pathways to pyrite [4, 5], which may be important in hydrothermal deep-sea vents [6]. The crystal chemistry of greigite resembles those of several modern-day key-enzymes active sites involved in iron-sulfur metabolism [7] and it has been shown that greigite is a key catalyst for the dynamics of carbon in hydrothermal systems [8]. Beyond the specific case of greigite, it has been suggested that iron sulfide minerals, found in the chimney cavities of hydrothermal vents [3], could have catalyzed CO_2_ reduction, forming a primitive acetyl CoA pathway [9, 10]. There is increasing interest in greigite due to the potential promising applications of this mineral in medicine, solid-state physics and chemistry [11]. Thus many efforts have been made to synthesize and control properties of greigite nanocrystals [12]. It has been known for a long time that magnetotactic bacteria biomineralize greigite and magnetite intracellularly [13]. Magnetite can also be biomineralized extracellularly by numerous prokaryote species [14, 15] and greigite associated to mackinawite is commonly reported in cultures of sulfate-reducing bacteria (SRB) [16, 17]. The accumulation of sulfide is required for the production of greigite [16]. However, it was not known whether sulfur-reducing hyperthermophilic heterotrophic microorganisms could also induce the production of greigite by external biomineralization processes. For instance, one could imagine that these hyperthermophilic organisms thriving within the pores of hydrothermal chimneys could induce greigite biomineralization which in turn would have major geochemical impacts in these hydrothermal systems. Moreover, the biomineralization of specific mineral phases might form the basis of biosignatures [18, 19]. To date, no hydrothermal biosignature of organisms inhabiting the hottest parts of hydrothermal vents has been yet discovered.

To test the hypothesis that hyperthermophilic anaerobic archaea of the *Thermococcales* order, the predominant hyperthermophilic organisms inhabiting hot parts of hydrothermal deep-sea vents [20], could influence the formation of iron sulfide minerals, in laboratory conditions, living cells were incubated in mineralization media containing ferrous iron, a major solute in the hydrothermal fluid [21]. We first used *Thermococcus prieurii* as a model organism, because this strain produces sulfur-rich vesicles which could favor the biomineralization processes [22] and then extended the study to other *Thermococcales*.

## Materials and methods

### Hyperthermophilic archaeal strains and growth conditions

*Thermococcus prieurii*, *T*. *nautili* and *T*. *kodakaraensis* were cultivated at 85°C with shaking, in Ravot medium modified which contains, per litre of distilled water: 1 g NH_4_Cl, 0.2 g MgCl_2_. 6H_2_O, 0.1 g CaCl_2_ . 2H_2_O, 0.1 g KCl, 0.83 g CH_3_COONa . 2H_2_O, 20 g NaCl, 1 g yeast extract, 3 g piperazine- N,N′-bis (2-ethanesulfonic acid) (PIPES buffer) and 0.001 g resazurin. The pH was adjusted to 7 before autoclaving. After autoclaving, the following sterile solutions were added aseptically: 5 ml of 6 % (w/v) K_2_HPO_4_ solution and 5 ml of 6 % (w/v) KH_2_PO_4_ solution. The medium was then dispensed (50 ml) into 100 ml sterile flasks and supplemented with 1 g (w/v) elemental sulfur l ^−1^. Anaerobiosis was obtained by applying a vacuum to the medium and saturating it with dinitrogen. Finally, a sterile solution of Na_2_S . 9H_2_O [final concentration 0.05 % (w/v)] was added to reduce the medium. Cells of *Thermococcales* in early stationary phase, with final concentrations of 5.10^7^ cells mL^-1^, were transferred to anoxic mineralization medium composed of ferrous sulfate (FeSO_4_) at a final concentration of 5 mM. Cultures were then incubated at 85°C with shaking, and were regularly sampled until 192 hours. This procedure retains some important characteristics of natural deep-sea vents (high Fe(II) concentration, high sulfur and sulfide thermodynamic activities, high temperature) but not all (for example no high hydrostatic pressure). We used a combination of scanning electron microscopy (SEM), scanning transmission electron microscopy (STEM), transmission electron microscopy (TEM), high-resolution transmission electron microscopy (HRTEM), cryo-electron microscopy (Cryo-EM), and Energy dispersive X-Ray spectroscopy (EDXS) to analyze the composition of the mineralized cells.

### Transmission electron microscopy (TEM), high-resolution TEM (HRTEM), scanning transmission electron microscopy (STEM) and scanning electron microscopy (SEM)

To prepare samples for transmission electron microscopy (TEM), high-resolution TEM (HRTEM) and scanning transmission electron microscopy (STEM), 2 ml cultures (at different times of mineralization: 5h, 10h, 24h, 48h, 72h, 96h, 120h, 144h, 168h, 192h) were centrifuged at 5000 x g for 20 min. The pellets were washed with 1 ml of TE (10 mM Tris-HCl, 1 mM EDTA) and were centrifuged at 1500 x g for 5 min to remove cellular debris. Supernatants were subsequently centrifuged at 5000 x g for 10 min. The pellets were then resuspended in 200 μl of TE. 20 μl droplets of samples were adsorbed onto a carbon-coated copper grid for 1 min. After removing the excess liquid, the grids were rinsed with sterile water. To prepare samples for scanning electron microscopy (SEM), cells were fixed in 2% glutaraldehyde in TE (10 mM Tris-HCl, 1 mM EDTA) for 4 h at 4°C, washed overnight TE. The cells were then post-fixed for 1 h at room temperature with 1% osmium tetroxide in TE. The cells were dehydrated in a graded ethanol series and dried by critical point drying with a Leica EM CPD300 instrument. For TEM, HRTEM and STEM, specimens were respectively examined using a JEOL JEM-100 CX II, operating at 120 kV and a JEOL JEM-2100F, equipped with a field effect gun operating at 200 kV; for SEM, specimens were observed with a Zeiss Gemini 1550VP field-emission scanning electron microscope (Ultra55) equipped with Everhart-Thornley secondary electron detectors, in-lens and backscattered electron detectors.

### Cryo-electron microscopy (Cryo-EM)

To prepare samples for Cryo-EM, 10 ml cultures were centrifuged at 5000 x g for 20 min. The pellets were resuspended with 50 μl of buffer (10 mM Tris-HCl, 100 mM NaCl, 5 mM CaCl_2_). 10 μl droplets of preparations were adsorbed onto a holey grid covered with a fine layer of carbon (QUANTIFOIL®, R2/4). After removing the excess liquid with Whatman® paper, the grids were quickly immersed in liquid ethane and transferred under liquid nitrogen into the microscope using a side entry nitrogen-cooled cryoholder (Gatan, 626-DH cryotransfer system). The observations were performed with a JEOL electron microscope-2100 transmission electron microscope with an acceleration voltage of 200 kV, at a nominal magnification of 10000 k. Images were recorded under low dose conditions with ultra-scan 1000 camera (Gatan, 2x2).

### Energy dispersive X-Ray Spectroscopy (EDXS) analysis

In the TEM, EDXS (JEOL, resolution of about 140eV/channel) was carried out using a Si(Li) detector with ultrathin polymer window. In the SEM, a silicon drift technology detector from Brucker with polymer window (resolution of 123 eV/channel at 100 kcounts/sec) was used.

## Results and discussion

### Mineralization of *Thermococcales* cells

**Fig 1:**
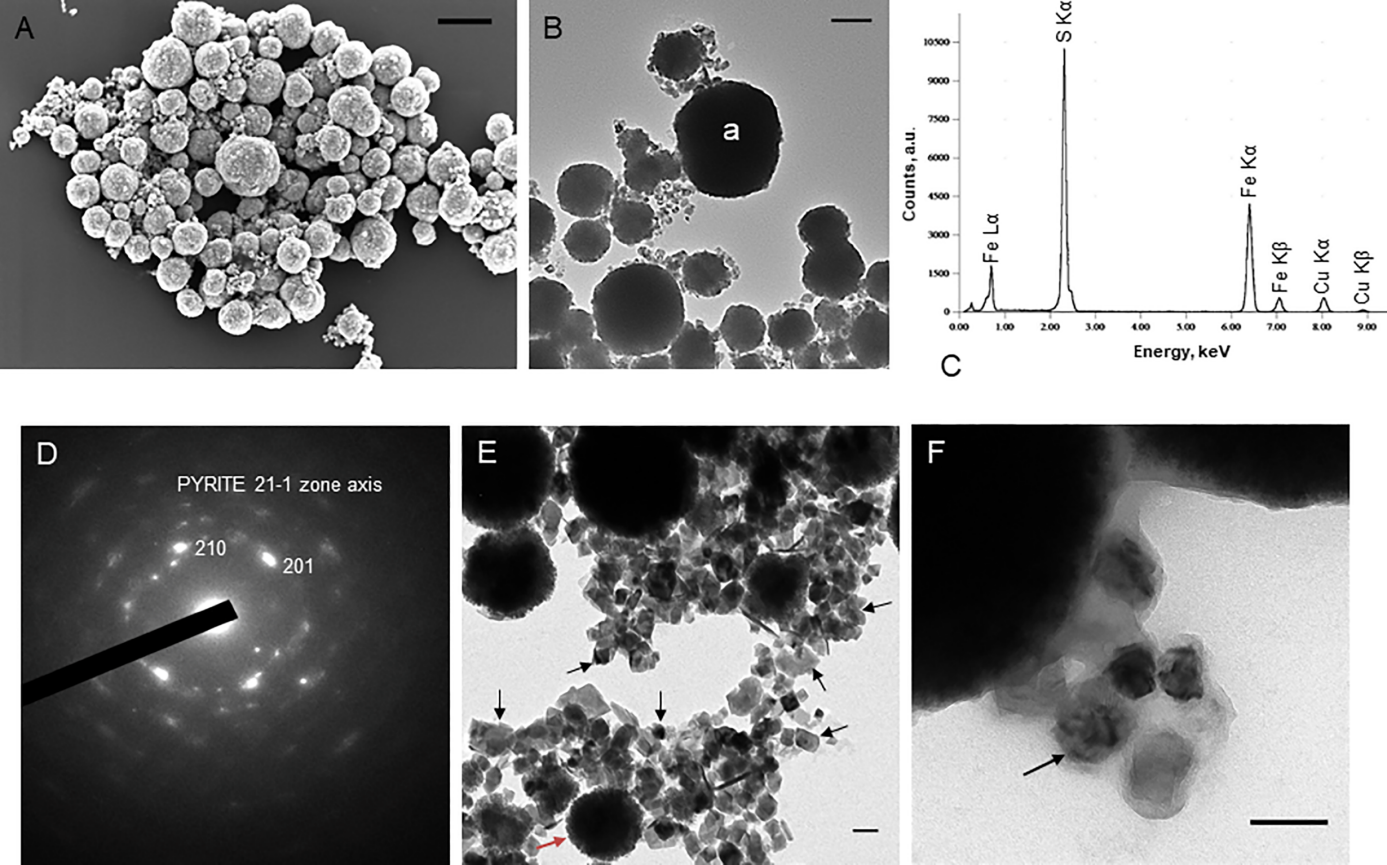
The iron sulfide mineralization of *Thermococcales* cells and vesicles (MVs). (A) Scanning electron microscopy image of *T*. *prieurii* cells and vesicles mineralized and organized in aggregates. Every sphere observed can be a mineralized cell (from 0.8 μm to 1.4 μm) or a vesicle (up to 0.5 μm). Scale bar = 1μm. (B)Transmission electron microscopy image of *T*. *prieurii* cells and vesicles entirely mineralized incubated for 168 hours with mineralization medium (“a” indicates the location of the EDXS analysis); scale bar = 500nm; (C) and elemental analysis by EDXS; with Fe/S ratio of about 0.5. Cu peaks are derived from the supporting grid. (D) Electron diffraction pattern of the minerals of the cell surfaces and/or filling, corresponding to pyrite. This is a polycrystalline pattern with strong preferred orientations toward a common 2–11 zone axis. (E) Transmission electron microscopy image of numerous extracellular greigite nanocrystals (indicated by black arrows) located near a vesicle containing pyrite (indicated by a red arrow); scale bar = 100nm (F) and near a cell; about fifty nanocrystals were counted to obtain the 30–70 nm range; scale bar = 100nm.

The incubation at 85°C of *Thermococcus* cells, in a medium containing ferrous sulfate (FeSO_4_), generated a black precipitate in the flasks, whereas no such precipitate was observed in the control growth medium without cells. This phenomenon is due the production of hydrogen sulfide (H_2_S) by *Thermococcales* upon reduction of sulfur during growth [20]. In presence of dissolved Fe^2+^, hydrogen sulfide is known to trigger the precipitation of iron sulfides (usually mackinawite, but also greigite and pyrite) [23]. After incubation, we indeed observed deposits of dense precipitates on the cell surfaces. Fig 1 shows *T*. *prieurii* cells and their membrane vesicles organized in aggregates (Fig 1A) and entirely covered and/or filled with dark precipitates (Fig 1B), which were shown by x-ray energy-dispersive spectroscopy (EDXS) to be iron sulfides (Fig 1C). On the basis of electron diffraction patterns, pyrite was identified unambiguously (Fig 1D). Another mineralization also occurred in the immediate vicinity of cells and vesicles (Fig 1E) as irregular and cuboidal extracellular crystals ranging from 30 nm to 70 nm (Fig 1F).

### Extracellular production of greigite

**Fig 2:**
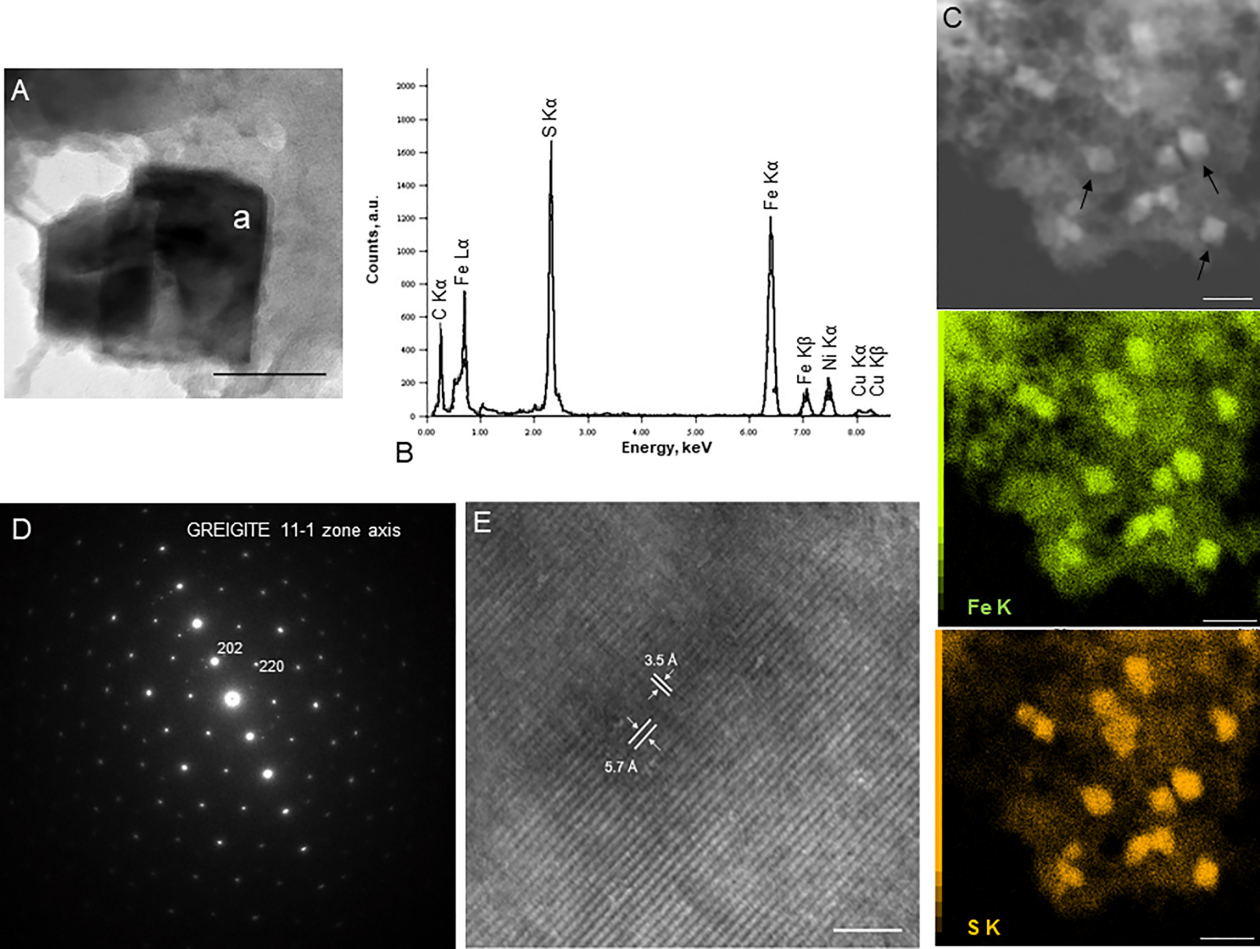
Nanocrystals of Fe_3_S_4_ greigite. (A) Transmission electron microscopy image of greigite nanocrystals. Scale bar = 50nm. (“a” indicates the location of the EDXS analysis) (B) and elemental analysis by EDXS; with Fe/S ratio of about 0.75. Cu peaks are derived from the supporting grid. (C) Scanning transmission electron microscopy image of greigite nanocrystals (indicated by black arrows) dispersed in mineralized extracellular polymeric substances and associated Fe (middle panel) and S maps (lower panel). Scale bar = 200nm. (D) Electron diffraction of a greigite crystal. (E) High-resolution transmission electron microscopy image of a greigite nanocrystal. The inter-reticular distances at 5.7 and 3.5 Angstrom correspond respectively to {111} and {220} sets of lattice planes; the image thus has a <110> zone axis. Scale bar = 5nm.

The iron sulfide nanocrystals (Fig 1D, Fig 1E, Fig 2), unambiguously characterized by electron diffraction (Fig 2C) and HRTEM (Fig 2D) as greigite, were visible after 96 hours of incubation. They continuously precipitated as we observed more crystals after 192 hours of incubation of *Thermococcales* cells in mineralization medium. Greigite was never observed in mineralization medium without cells. In order to evaluate the role of *Thermococcales* cells in this mineralization step, mineralization processes were modeled using the CHESS geochemical software [24]. Models of the mineralization medium used in this study showed that the iron sulfide minerals precipitated are pyrite (S1 Fig). The formation of greigite was exclusively achieved when precipitation of pyrite was disabled (S2 Fig). Pure (i.e. not intimately mixed with pyrite nor mackinawite nor pyrrhotite) greigite nanocrystals were detected in the immediate vicinity of the *Thermococcales* cells. More than thirty diffraction patterns (powder or single crystal patterns) were indexed into greigite and could not be indexed as mackinawite nor pyrrhotite nor pyrite. EDXS data are only semi-quantitative but they still allow distinguishing greigite (Fe/S nominal ratio of 0.75) from pyrite (Fe/S of 0.5).

### An original mechanism

**Fig 3:**
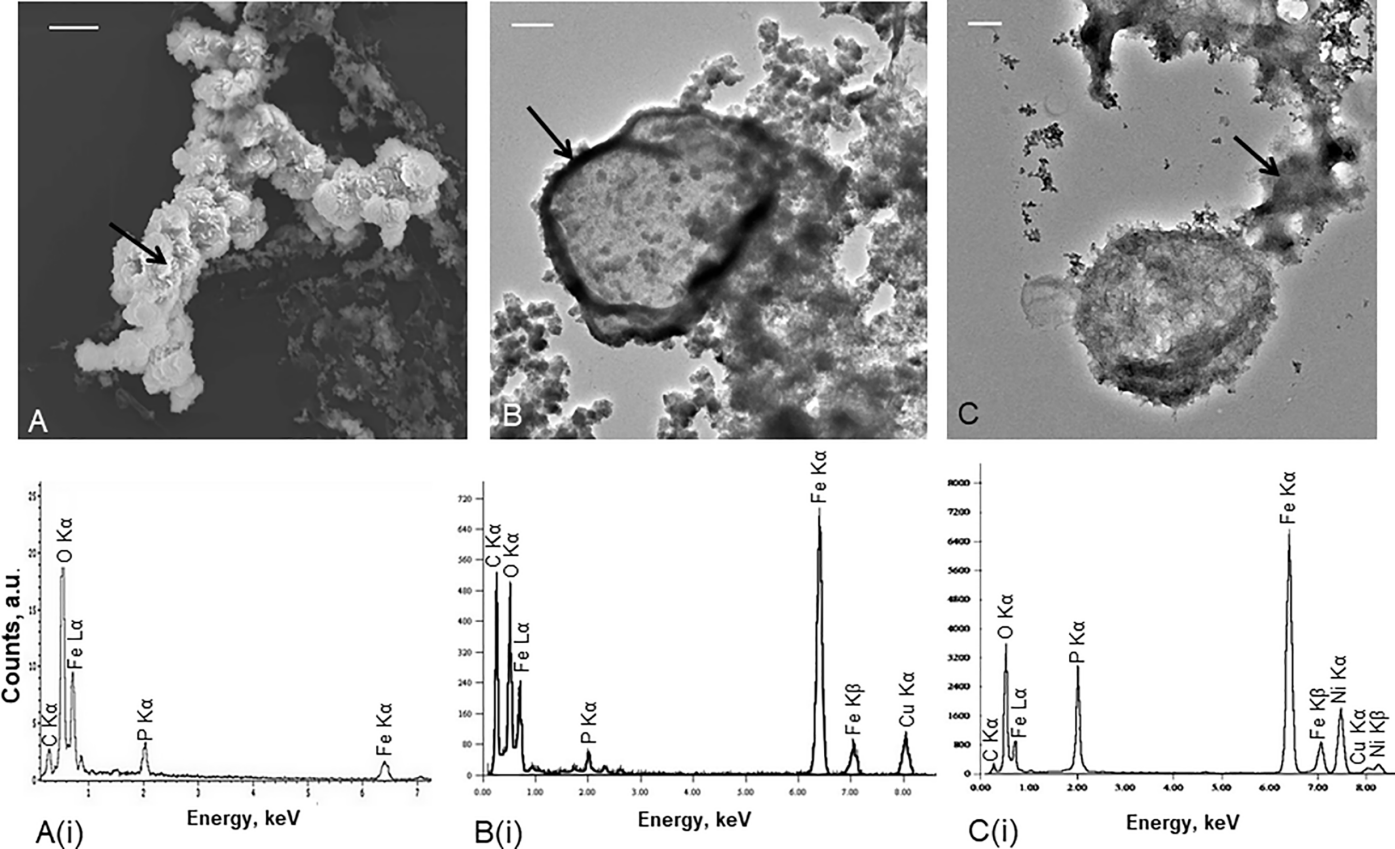
The first stages of greigite mineralization of *Thermococcales*. (A) Scanning and transmission electron microscopy images of *T*. *prieurii* cells incubated for 5 hours; scale bar = 2μm, (B) 24 hours; scale bar = 200nm and (C) 48 hours; scale bar = 100nm with mineralization medium and their respective elemental analyses by EDXS (A(i), B(i), C(i)). Cu and Ni peaks are derived from the supporting grid. For each microscopy images, the black arrow indicates the location of the EDXS analysis. The first minerals formed on the cell surfaces correspond to amorphous Fe(III) phosphates (A, B). The *Thermococcales* S-layer is the highly reactive interface for iron sequestration (B). After 48 hours of incubation in mineralization medium, *Thermococcales* cells release extracellular polymeric substances mineralized with amorphous iron phosphates (C).

Greigite often converts to pyrite when an excess of H_2_S is present [4, 25]. It was therefore interesting that, despite an excess of H_2_S in the media, we observed greigite in all investigated samples, both fresh and old specimens, over a period ranging from a few days to several weeks. This suggests that this transient mineral phase remains relatively stable in the proximity of *Thermococcales*. Another iron-bearing phase, iron phosphate was observed, but only in short time experiments (from 5 hours to 48 hours of incubation in mineralization medium) (Fig 3). This phase was seen as amorphous in selected area diffraction patterns; semi-quantitative EDXS analyses provide a constant and reproducible ratio Fe/P, close to 1. These data point to an amorphous Fe(III) phosphate (Fig 3A(i)) possibly hydrated. In case of an Fe(II) phosphate instead of Fe(III), the Fe/P ratio would rather be of 1.5. The addition of OH^-^ groups would also increase the Fe/P ratio above 1 making their presence very unlikely. From 5 hours to 48 hours of incubation, *T*. *prieurii* cells were exclusively associated with these Fe(III) phosphates (Fig 3A to Fig 3C).

**Fig 4:**
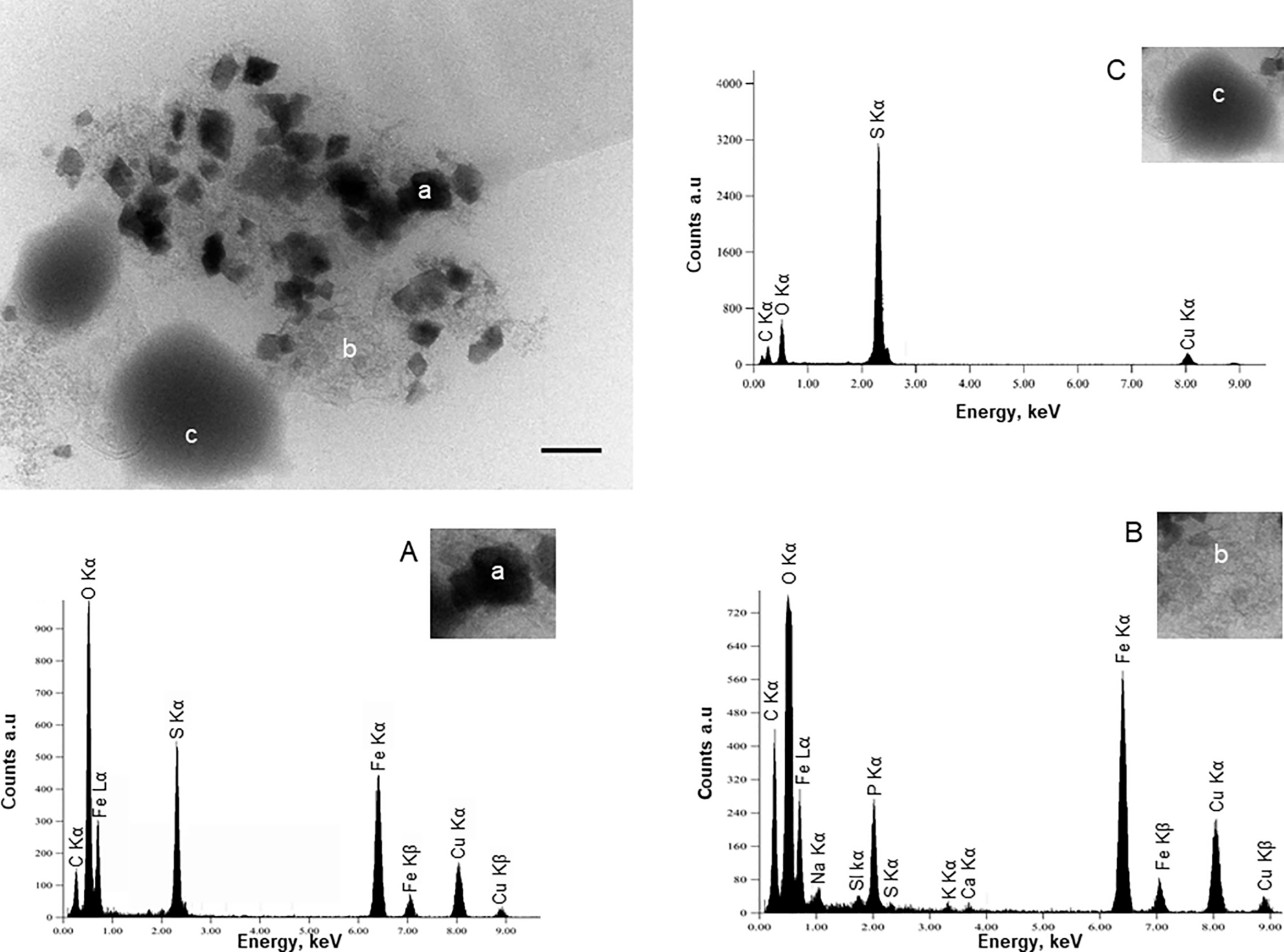
The phosphate to greigite mineralization process. Transmission electron microscopy image; scale bar = 100nm; and EDXS spectra of nanocrystals of greigite (a) dispersed in the organic matrix mineralized with amorphous Fe(III) phosphates (b) located near the sulfur vesicles of *T*. *prieurii* (c). The lettering indicates the location of the spot scan for the EDXS analyses. (A) EDXS spectrum of a greigite crystal showing iron and sulfur peaks; Fe/S ratio = 0.75. (B) EDXS spectrum of the organic matrix loaded with iron phosphates. (C) EDXS spectrum of a sulfur-rich vesicle. Cu peaks are derived from the supporting grid.

Archaeal surfaces may favor interactions between the cells and iron [26]. Usually, *Thermococcales* S-layer exposes negative charges favoring iron adsorption [27]. It has recently been confirmed that the archaeal S-layer, independently of cellular metabolic processes, is a favorable site for Fe(III) phosphate formation [28]. Fig 3B shows that *Thermococcales* cell surface was a highly reactive interface for iron sequestration and nucleation of Fe(III) phosphates (Fig 3B(i)). Most mineralized *Thermococcales* cells conserved their whole structure until 24 hours of mineralization. After 48 hours of incubation many dying or dead cells had released and/or excreted cytoplasmic organic matter including phosphate which is known to come from cellular degradation [29]. This process resulted in the production of extracellular polymeric substances (EPS), possibly remnants of excreted S-layers, on which amorphous Fe(III) phosphates nucleated (Fig 3C and Fig 3C(i)). A phosphate response of cells to metal cation stress is commonly observed in numerous cases [30] even in nominally phosphate-free experiments as it was the case in some of our experiments. The EPS loaded with Fe(III) phosphates provided substrates for nucleation of greigite nanocrystals. Greigite was indeed formed at the expense of the Fe(III) phosphates on the EPS substrate (Fig 4) confirming the fact that iron phosphate appears in short term experiments and is then fully replaced by greigite in longer term experiments.

**Fig 5:**
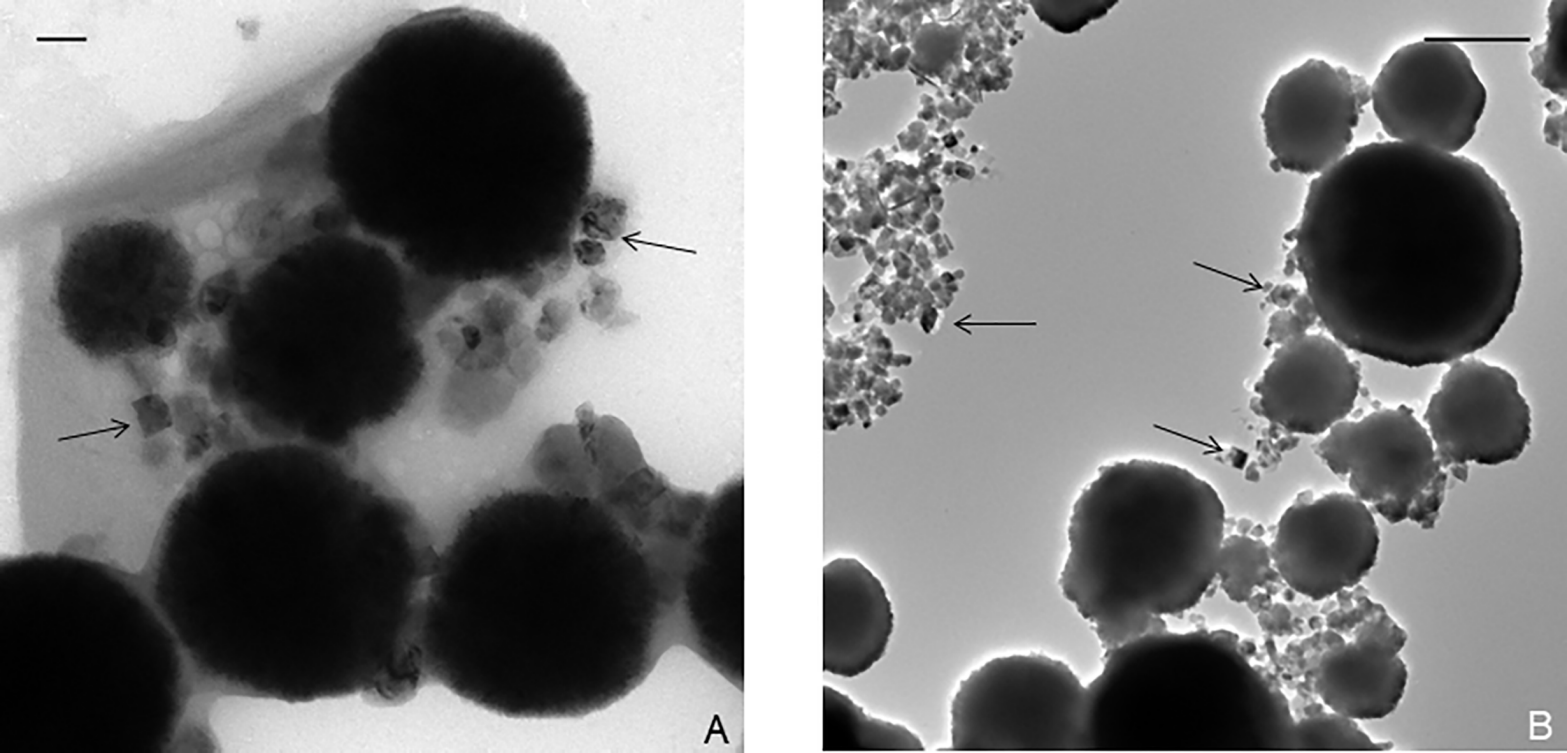
The production of greigite by other *Thermococcales*. (A) Transmission electron microscopy image of *T*. *nautili* cells mineralized incubated for 168 hours with mineralization medium. Scale bar = 100nm. (B) Transmission electron microscopy image of *T*. *kodakaraensis* cells mineralized incubated for 168 hours with mineralization medium. Scale bar = 500nm. The nanocrystals of greigite are indicated by black arrows.

*Thermococcales* are known to produce a large number of membrane vesicles (MVs) [31]. Interestingly, a previous study showed that *T*. *prieurii* produces sulfur vesicles (SVs, around 4 to 16 SVs per cell) containing native sulfur or polysulfides [22]. TEM observations showed that greigite nanocrystals were located near MVs filled with pyrite nanocrystals (Fig 1E). In presence of Fe(II) from the mineralization medium, most SVs were converted to MVs containing FeS_2_. However, some SVs were still observed (Fig 4C) located near the greigite crystals, as shown in Fig 4 (Fig 4A). The greigite nanocrystals are dispersed in the EPS loaded with Fe(III) phosphates (Fig 4B). The presence of initial Fe(III) as Fe(III) phosphate favors the formation of greigite which contains one Fe(II) and two Fe(III) per formula [32]. In order to investigate the degree of generality of this mechanism, two other *Thermococcus* species were considered. The closely related archaeal species *T*. *kodakaraensis* and *T*. *nautili* behave differently when it comes to production of sulfur vesicles (SVs). Whereas *T*. *kodakaraensis* produces less SVs (around 2 SVs per cell) than *T*. *prieurii*, *T*. *nautili* produces no SVs at all [22]. But after 168 hours of incubation, the nanocrystals in the close vicinity of cells in both types of *T*. *kodakaraensis* and *T*. *nautili* were found to be greigite (Fig 5A and Fig 5B). Iron phosphates were also observed for shorter incubation times. Although it seems that the production of sulfur vesicles does not strongly affect the mineralization process, the presence of SVs could influence the time required for growth of the greigite. The first greigite produced by *T*. *prieurii* and *T*. *kodakaraensis*, respectively, are visible after 96 hours and 120 hours of incubation whereas greigite formed by *T*. *nautili* are exclusively observed after 168 hours of incubation.

The main point is thus that under strong mineralization conditions (high Fe(II) concentrations in a sulfidic medium nominally free of phosphates), *Thermococcales* produce EPS loaded with Fe(III) phosphate which at these temperatures evolves rapidly into greigite. This mechanism is quite different from biologically controlled synthesis of greigite by magnetotactic bacteria (MTB) and biologically induced synthesis of greigite by sulfate reducing bacteria (SRB). In both these cases, the greigite phase is formed by the partial oxidation of mackinawite (FeS) [16, 25]. More recently, it has been shown that the methanogen *Methanocaldococcus jannaschii* produces greigite and pyrite when hematite is added to the growth medium [33] because greigite usually grows on the surface of other iron-bearing minerals [34]. It would be interesting to prospect whether the iron phosphate mechanism that we observe in the present study could operate to some extent in greigite produced by MTB, SRB or in *Methanocaldococcus jannaschii*. Greigite production implies that two thirds of the initial Fe(II) which is the only iron species added under anoxic conditions in ours experiments was oxidized into Fe(III). This oxidation step occurs when the Fe(III) phosphate intermediate phase grows on EPS. The oxidizing agent could be either residual O_2_ or oxidized functional groups in the EPS (e.g. carboxylic groups) or native sulfur or polysulfides or even H_2_S. Then preservation of greigite [35] depends on the balance between the abundance of reactive iron, accumulated on the cell surfaces and/or on extracellular polymeric substances, and on the sulfide or polysulfide activities. In the long term experiments and in hydrothermal systems, greigite, which is an intermediate on the way to pyrite [4, 5], might eventually transform to pyrite and be a transient phase continuously formed due to *Thermococcales* metabolism and thus maintained at relatively low levels. Due to its recognized catalytic activity [8] and to its nanometric size leading to large reactive surfaces, greigite produced by *Thermococcales* metabolism will influence carbon species and carbon cycle in hydrothermal chimneys. Moreover, the formation of greigite (an Fe(III) bearing sulfide) and of pyrite (an Fe(II) disulfide) from the Fe(II) and sulfide dominated speciation in hydrothermal vents requires consumption of an oxidant. The original mechanism discovered in this study could thus participate to consumption of oxidative power by hydrothermal chimneys at the interface between the reduced hydrothermal fluid and the oxidized seawater. It has already been reported that the production of biominerals could provide some benefits to the microbial cells [36]. In the fluctuating environment of a hydrothermal chimney, this mechanism could contribute to protect the cellular components from toxic oxygen and/or toxic oxygen radicals.

The search of mineralogical biosignatures is a great challenge to establish that life is present or has existed in a particular geological environment [18]. In hydrothermal vents, tubes and filaments of iron oxyhydroxide minerals have been explained as remains of iron-metabolizing filamentous bacteria [19, 37, 38]. These particular mineral assemblages are considered as biosignatures and were recently proposed as evidences for biological activity in the oldest hydrothermal vent precipitates [39]. However, no evidence of *Thermococcales* which are recognized major inhabitants of the hot parts of chimneys have been yet observed. In hydrothermal deep–sea vents, the observation of greigite or alternatively of pyrites structurally recognizable as having evolved from the greigite nanocrystals associated to then silicified ancient EPS could be explained as remains of sulfur-reducers microorganisms as *Thermococcales*. The greigite nanocrystals formed seem common to most *Thermococcales* since all species tested so far have been able to produce greigite.

Our results suggest that *Thermococcales* have the potential to biomineralize greigite in their habitats. This ability makes these organisms possible influential agents in the geochemistry of carbon, iron and sulfur cycles in hydrothermal deep-sea vents and renders them possible actors of the consumption of oxidative power by these biogeochemical systems.

## Supporting information

S1 Fig Calculated mineralization of pyrite using our mineralization conditions (Neutral pH, Temperature of 85°C, excess of sulfur). Results of modeling by using Chess software (version: 3.9.6).
(DOCX)

S2 Fig Calculated mineralization of greigite in the same previous mineralization conditions with restriction to precipitate pyrite. Results of modeling by using Chess software (version: 3.9.6).
(DOCX)
