# Supplementary material for: Greigite nanocrystals produced by hyperthermophilic archaea of Thermococcales order
Source: PLoS One. 2018 Aug 2;13(8):e0201549. doi: 10.1371/journal.pone.0201549 (PMC6072027; doi:10.1371/journal.pone.0201549)
Supplement: S1 Fig — Results of modeling by using Chess software (version: 3.9.6). (DOCX) [file pone.0201549.s001.docx]

**S1 Fig**

=============================================================================

CHESS General Report

========================

CHESS ver. : 3.9.6

Session date : Mon Jul 11 12:45:49 2016

This file : /Users/francoisguyot/Desktop/Projet/Ete16/Article/Aurore/Calcul Chess/outputbiominpyrite.out

Database file : /Applications/QChess/AQChess_lv1_Thermoddem_15Dec2011.tdb

Activity model: truncated-davies

EOS model: perfect

Interface model: surface-complexation, double-layer

57 species were considered for the equilibrium calculations

51 solids were considered for the equilibrium calculations

==============================================================================

calculating initial equilibrium...

...converged in 31 iterations...

dissolving S(alpha)...converged in 7 iterations...

precipitating Fe11S12, saturation index = 88.8389

...converged in 15 iterations...

precipitating Pyrite, saturation index = 6.98735

dissolving Fe11S12...converged in 24 iterations...

...success!

Final equilibrium of the main solution:

=======================================

pH : 7

ionic strength : 0.523275

temperature : 85 Celsius

redox potential (Eh) : -0.33365 volts

electron activity (pe) : -4.6938

electrical imbalance : -0.00013336 eq/l

carbonate alkalinity : 0 eq/l

solvent activity : 1

solvent mass : 1000 g

total dissolved solids : 36.729 g/kg

solution mass : 1036.7 g

solution density : 1013.4 g/l

solution volume : 1.023 liter

mineral volume : 0.0001197 liter

system pressure : 1 atm

Aqueous species:

---------------- ---molal---- ---mol/l---- ----ppm---- ---g/l----

Na[+] 0.47063 0.46004 10820 10.576

HS[-] 0.29406 0.28744 9725.6 9.5067

H2S 0.073504 0.07185 2505.2 2.4488

NaSO4[-] 0.07151 0.069901 8513.5 8.3219

SO4[2-] 0.050089 0.048962 4811.7 4.7035

S5[2-] 0.00127 0.0012414 203.61 0.19903

S4[2-] 0.00081069 0.00079245 103.98 0.10164

S3[2-] 0.00033931 0.00033168 32.641 0.031907

NaS2O3[-] 4.9539e-05 4.8425e-05 6.6937 0.0065431

S2O3[2-] 4.8452e-05 4.7362e-05 5.4329 0.0053107

S2[2-] 1.6933e-05 1.6552e-05 1.086 0.0010615

OH[-] 4.1339e-06 4.0409e-06 0.070306 6.8724e-05

HSO4[-] 1.3859e-06 1.3547e-06 0.13453 0.0001315

NaOH 2.3382e-07 2.2856e-07 0.0093521 9.1416e-06

H[+] 1.3617e-07 1.3311e-07 0.00013725 1.3417e-07

H2(aq) 1.975e-08 1.9306e-08 3.9814e-05 3.8918e-08

S[2-] 5.4535e-09 5.3308e-09 0.00017487 1.7094e-07

HS2O3[-] 2.6845e-10 2.6241e-10 3.0372e-05 2.9689e-08

HSO3[-] 2.209e-10 2.1593e-10 1.7909e-05 1.7506e-08

SO3[2-] 1.8578e-10 1.816e-10 1.4874e-05 1.4539e-08

H2SO3 6.5094e-15 6.3629e-15 5.3429e-10 5.2227e-13

H2S2O3 2.501e-16 2.4447e-16 2.8548e-11 2.7905e-14

S4O6[2-] 1.9141e-17 1.871e-17 4.2926e-12 4.196e-15

FeSO4 3.2931e-18 3.219e-18 5.0026e-13 4.8901e-16

Fe[2+] 1.1642e-18 1.138e-18 6.5017e-14 6.3554e-17

FeOH[+] 6.1747e-20 6.0357e-20 4.4985e-15 4.3973e-18

S2O5[2-] 1.7674e-21 1.7276e-21 2.5474e-16 2.49e-19

FeO 2.5372e-22 2.4801e-22 1.8229e-17 1.7819e-20

S2O4[2-] 6.8495e-23 6.6954e-23 8.7762e-18 8.5788e-21

FeO[+] 7.0829e-25 6.9235e-25 5.0888e-20 4.9743e-23

HFeO2(aq) 6.9451e-25 6.7889e-25 6.171e-20 6.0322e-23

Fe(OH)4[-] 5.6055e-25 5.4794e-25 6.9439e-20 6.7877e-23

FeHSO4[+] 4.9421e-25 4.8309e-25 7.5574e-20 7.3874e-23

Solids:

---------------- ---molal---- ---mol/l---- ----ppm---- ---g/l----

Pyrite 0.005 0.0048875 599.89 0.5864

Surfaces and volumes:

---------------- ---m2/mol--- ---m2/g---- ---m2/l---- ---liter---

Pyrite 200 1.667 0.9775 0.0001197

Cumulative concentrations (molal):

----------------------------------

aqueous mineral colloidal organic fixed

H[+] 0.45768 0.01 0 0 0

Na[+] 0.54219 0 0 0 0

Fe[2+] 4.5193e-18 0.005 0 0 0

SO4[2-] 0.5 0.01 0 0 0

O2(aq) 4.9085e-25 0 0 0 0

Cumulative concentrations (gram/l):

-----------------------------------

aqueous mineral colloidal organic fixed

H[+] 0.45093 0.0098526 0 0 0

Na[+] 12.184 0 0 0 0

Fe[2+] 2.4671e-16 0.27295 0 0 0

SO4[2-] 46.951 0.93902 0 0 0

O2(aq) 1.5353e-23 0 0 0 0

Potential fugacities of gases:

------------------------------

H2S(g) 2.0213

H2O(g) 0.57191

H2(g) 2.4442e-05

S2(g) 1.4436e-11

SO2(g) 2.4536e-14

Saturation indices (down to -3) of solids:

------------------------------------------

Pyrite 0

S(gamma) -0.31392

S(alpha) -0.32196

S(beta) -0.32303

Marcassite -0.59816

Thenardite -2.132

==============================================================================
