# Supplementary material for: Greigite nanocrystals produced by hyperthermophilic archaea of Thermococcales order
Source: PLoS One. 2018 Aug 2;13(8):e0201549. doi: 10.1371/journal.pone.0201549 (PMC6072027; doi:10.1371/journal.pone.0201549)
Supplement: S2 Fig — Results of modeling by using Chess software (version: 3.9.6). (DOCX) [file pone.0201549.s002.docx]

**S2 Fig**

==============================================================================

CHESS General Report

========================

CHESS ver. : 3.9.6

Session date : Wed Jul 6 11:36:02 2016

This file : /Users/francoisguyot/Desktop/Projet/Ete16/Article/Aurore/Calcul Chess/outputbiomin.out

Database file : /Applications/QChess/AQChess_lv1_Thermoddem_15Dec2011.tdb

Activity model: truncated-davies

EOS model: perfect

Interface model: surface-complexation, double-layer

Precipitation is disabled of

Troilite,

Pyrite,

Marcassite,

Fe10S11,

Fe11S12,

Fe7.016S8,

Fe9S10

57 species were considered for the equilibrium calculations:

-------------------------------------------------------------

name: mole-weight: name: mole-weight:

H2O 0.0180153 | H[+] 0.00100794

Na[+] 0.0229898 | Fe[2+] 0.055847

SO4[2-] 0.0960636 | O2(aq) 0.0319988

HS[-] 0.0330739 | S[2-] 0.032066

Fe[3+] 0.055847 | H2(aq) 0.00201588

HSO5[-] 0.113071 | S2O3[2-] 0.11213

S2O4[2-] 0.12813 | S2O6[2-] 0.160128

S2O8[2-] 0.192127 | SO3[2-] 0.0800642

H2(g) 0.00201588 | H2O(g) 0.0180153

H2S(g) 0.0340819 | O2(g) 0.0319988

S2(g) 0.064132 | SO2(g) 0.0640648

Fe(OH)4[-] 0.123876 | Fe(SO4)2[-] 0.247974

Fe2(OH)2[4+] 0.145709 | Fe2(SO4)3 0.399885

FeH(SO4)2 0.248982 | FeHSO4[+] 0.152919

FeHSO4[2+] 0.152919 | FeO 0.0718464

FeO[+] 0.0718464 | FeOH[+] 0.0728543

FeOH[2+] 0.0728543 | FeSO4[+] 0.151911

FeSO4 0.151911 | H2S 0.0340819

H2S2O3 0.114146 | H2S2O4 0.130145

H2SO3 0.0820801 | HFeO2(aq) 0.0888537

HFeO2[-] 0.0888537 | HS2O3[-] 0.113138

HS2O4[-] 0.129138 | HSO3[-] 0.0810721

HSO4[-] 0.0970715 | NaOH 0.0399971

NaS2O3[-] 0.13512 | NaSO4[-] 0.119053

OH[-] 0.0170073 | S2[2-] 0.064132

S2O5[2-] 0.144129 | S3[2-] 0.096198

S3O6[2-] 0.192194 | S4[2-] 0.128264

S4O6[2-] 0.22426 | S5[2-] 0.16033

S5O6[2-] 0.256326

51 solids were considered for the equilibrium calculations

-------------------------------------------------------------

name: mole-weight: name: mole-weight:

S(alpha) 0.032066 | Copiapite 1.24994

Coquimbite 0.562022 | Erdite 0.178999

Fe(element) 0.055847 | Fe(OH)2 0.0898617

Fe10S11 0.911196 | Fe11S12 0.999109

Fe2(SO4)3 0.399885 | Fe7.016S8 0.648351

Fe9S10 0.823283 | FeO 0.0718464

Ferricopiapite 1.24893 | Ferrihydrite(2L) 0.106869

Ferrihydrite(6L) 0.106869 | Ferrohexahydrite 0.260002

FeS(am) 0.087913 | FeSO4 0.151911

Goethite 0.0888537 | Greenrust(OH) 0.267569

Greenrust(SO3) 0.871019 | Greenrust2(SO4) 0.671264

Greigite 0.295805 | Hematite 0.159692

Jarosite(H) 0.480735 | Jarosite(Na) 0.484702

Kornelite 0.525992 | Lepidocrocite 0.0888537

Mackinawite 0.087913 | Maghemite(disordered) 0.159692

Magnetite 0.231539 | Magnetite(am) 0.231539

Marcassite 0.119979 | Melanterite 0.278018

Mirabilite 0.322196 | Na(element) 0.0229898

Na2O 0.0619789 | Na2SO4.FeSO4:4H2O 0.366015

NaFeS2 0.142969 | Pyrite 0.119979

Pyrrhotite 0.087913 | Romerite 0.804009

Rozenite 0.223972 | S(beta) 0.032066

S(gamma) 0.032066 | Schwertmannite 0.917001

Siderotil 0.241987 | Szomolnokite 0.169926

Thenardite 0.142043 | Troilite 0.087913

Wustite 0.0688865

==============================================================================

calculating initial equilibrium...

...converged in 31 iterations...

dissolving S(alpha)...converged in 7 iterations...

precipitating Greigite, saturation index = 21.4593

...converged in 14 iterations...

...success!

Final equilibrium of the main solution:

=======================================

pH : 7

ionic strength : 0.526378

temperature : 85 Celsius

redox potential (Eh) : -0.33366 volts

electron activity (pe) : -4.694

electrical imbalance : -5.0255e-05 eq/l

carbonate alkalinity : 0 eq/l

solvent activity : 1

solvent mass : 1000 g

total dissolved solids : 36.972 g/kg

solution mass : 1037 g

solution density : 1013.6 g/l

solution volume : 1.023 liter

mineral volume : 0.0001485 liter

system pressure : 1 atm

Aqueous species:

---------------- ---molal---- ---mol/l---- ----ppm---- ---g/l----

Na[+] 0.47358 0.46293 10888 10.643

HS[-] 0.29588 0.28922 9786 9.5658

H2S 0.073961 0.072297 2520.7 2.464

NaSO4[-] 0.072154 0.07053 8590.1 8.3969

SO4[2-] 0.050225 0.049095 4824.8 4.7163

S5[2-] 0.0013054 0.001276 209.29 0.20458

S4[2-] 0.00082886 0.00081021 106.31 0.10392

S3[2-] 0.00034508 0.00033731 33.196 0.032449

NaS2O3[-] 5.0296e-05 4.9164e-05 6.796 0.0066431

S2O3[2-] 4.8886e-05 4.7786e-05 5.4815 0.0053582

S2[2-] 1.7129e-05 1.6744e-05 1.0985 0.0010738

OH[-] 4.1339e-06 4.0409e-06 0.070306 6.8724e-05

HSO4[-] 1.3896e-06 1.3584e-06 0.1349 0.00013186

NaOH 2.3528e-07 2.2999e-07 0.0094107 9.1989e-06

H[+] 1.3617e-07 1.3311e-07 0.00013725 1.3417e-07

H2(aq) 1.9767e-08 1.9322e-08 3.9848e-05 3.8952e-08

S[2-] 5.4874e-09 5.3639e-09 0.00017596 1.72e-07

HS2O3[-] 2.7086e-10 2.6476e-10 3.0644e-05 2.9955e-08

FeSO4 2.6553e-10 2.5956e-10 4.0337e-05 3.943e-08

HSO3[-] 2.217e-10 2.1671e-10 1.7974e-05 1.7569e-08

SO3[2-] 1.8644e-10 1.8225e-10 1.4927e-05 1.4592e-08

Fe[2+] 9.3618e-11 9.1511e-11 5.2283e-06 5.1106e-09

FeOH[+] 4.9653e-12 4.8535e-12 3.6174e-07 3.536e-10

FeO 2.0403e-14 1.9943e-14 1.4658e-09 1.4329e-12

H2SO3 6.5328e-15 6.3858e-15 5.3621e-10 5.2415e-13

H2S2O3 2.5234e-16 2.4666e-16 2.8803e-11 2.8155e-14

FeO[+] 5.6931e-17 5.565e-17 4.0903e-12 3.9983e-15

HFeO2(aq) 5.5824e-17 5.4568e-17 4.9602e-12 4.8486e-15

Fe(OH)4[-] 4.5056e-17 4.4043e-17 5.5814e-12 5.4558e-15

FeHSO4[+] 3.9849e-17 3.8953e-17 6.0937e-12 5.9566e-15

S4O6[2-] 1.9468e-17 1.903e-17 4.366e-12 4.2677e-15

HFeO2[-] 1.1625e-17 1.1363e-17 1.0329e-12 1.0096e-15

FeOH[2+] 1.9238e-21 1.8805e-21 1.4016e-16 1.37e-19

S2O5[2-] 1.7801e-21 1.7401e-21 2.5657e-16 2.508e-19

Fe(SO4)2[-] 8.8932e-22 8.6931e-22 2.2053e-16 2.1557e-19

S2O4[2-] 6.9048e-23 6.7494e-23 8.8471e-18 8.648e-21

FeSO4[+] 9.4498e-24 9.2372e-24 1.4355e-18 1.4032e-21

FeH(SO4)2 6.2626e-26 6.1217e-26 1.5593e-20 1.5242e-23

Fe[3+] 1.3227e-26 1.2929e-26 7.3866e-22 7.2204e-25

HS2O4[-] 1.7383e-27 1.6992e-27 2.2449e-22 2.1943e-25

S2O6[2-] 8.4781e-28 8.2873e-28 1.3576e-22 1.327e-25

S3O6[2-] 5.2831e-30 5.1643e-30 1.0154e-24 9.9255e-28

S5O6[2-] 2.8921e-31 2.8271e-31 7.4133e-26 7.2465e-29

FeHSO4[2+] 1.3272e-31 1.2973e-31 2.0295e-26 1.9839e-29

H2S2O4 9.1271e-34 8.9218e-34 1.1879e-28 1.1611e-31

Fe2(OH)2[4+] 4.9016e-40 4.7914e-40 7.1421e-35 6.9814e-38

Fe2(SO4)3 3.132e-47 3.0615e-47 1.2524e-41 1.2242e-44

HSO5[-] 1.9052e-52 1.8623e-52 2.1542e-47 2.1058e-50

O2(aq) 5.6339e-61 5.5071e-61 1.8028e-56 1.7622e-59

S2O8[2-] 2.1461e-64 2.0978e-64 4.1232e-59 4.0304e-62

Solids:

---------------- ---molal---- ---mol/l---- ----ppm---- ---g/l----

Greigite 0.0016667 0.0016292 493.01 0.48192

Total concentrations*:

--------------------- ---molal---- ---mol/l---- ----g/l---- ---grams---

H2O 55.5 54.25 977.3 999.8

H[+] 0.4642 0.4537 0.4573 0.4678

Na[+] 0.5458 0.5335 12.27 12.55

Fe[2+] 0.005 0.004887 0.273 0.2792

SO4[2-] 0.51 0.4985 47.89 48.99

O2(aq) -0.77 -0.7527 -24.08 -24.64

------

*Note: these values are 'mathematical' total concentrations, as defined

by the methodology applied. They can be negative. See the technical

documents about CHESS for more detailed information on this matter.

Surfaces and volumes:

---------------- ---m2/mol--- ---m2/g---- ---m2/l---- ---liter---

Greigite 600 2.028 0.9775 0.0001485

Cumulative concentrations (molal):

----------------------------------

aqueous mineral colloidal organic fixed

H[+] 0.46083 0.0033333 0 0 0

Na[+] 0.54579 0 0 0 0

Fe[2+] 3.6414e-10 0.005 0 0 0

SO4[2-] 0.50333 0.0066667 0 0 0

O2(aq) 3.9454e-17 0 0 0 0

Cumulative concentrations (molar):

----------------------------------

aqueous mineral colloidal organic fixed

H[+] 0.45047 0.0032583 0 0 0

Na[+] 0.53351 0 0 0 0

Fe[2+] 3.5594e-10 0.0048875 0 0 0

SO4[2-] 0.49201 0.0065167 0 0 0

O2(aq) 3.8566e-17 0 0 0 0

Cumulative concentrations (gram/l):

-----------------------------------

aqueous mineral colloidal organic fixed

H[+] 0.45404 0.0032842 0 0 0

Na[+] 12.265 0 0 0 0

Fe[2+] 1.9878e-08 0.27295 0 0 0

SO4[2-] 47.264 0.62601 0 0 0

O2(aq) 1.2341e-15 0 0 0 0

Potential fugacities of gases:

------------------------------

H2S(g) 2.0338

H2O(g) 0.57191

H2(g) 2.4464e-05

S2(g) 1.459e-11

SO2(g) 2.4624e-14

O2(g) 7.2059e-58

Saturation indices (down to -300) of solids:

--------------------------------------------

Fe11S12 10.161

Fe10S11 9.1424

Fe9S10 9.0604

Fe7.016S8 8.7539

Pyrite 7.9103

Marcassite 7.3122

Greigite 0

Troilite -0.048341

Pyrrhotite -0.13073

Mackinawite -0.28731

S(gamma) -0.31161

S(alpha) -0.31965

S(beta) -0.32072

FeS(am) -0.74478

Erdite -1.4973

Thenardite -2.1254

Mirabilite -3.9054

NaFeS2 -4.5078

Goethite -4.6743

Lepidocrocite -5.8568

Fe(OH)2 -6.8553

FeO -6.8814

Ferrihydrite(6L) -6.9025

Wustite -7.0478

Ferrihydrite(2L) -7.209

Hematite -8.3267

Szomolnokite -9.3874

Siderotil -10.153

Ferrohexahydrite -10.199

Rozenite -10.313

Maghemite(disordered) -10.677

Melanterite -10.689

FeSO4 -11.247

Na2SO4.FeSO4:4H2O -11.947

Magnetite -12.767

Fe(element) -14.38

Magnetite(am) -16.29

Greenrust(OH) -25.913

Na(element) -34.629

Jarosite(H) -38.565

Jarosite(Na) -42.372

Na2O -44.134

Coquimbite -47.371

Kornelite -47.64

Fe2(SO4)3 -51.761

Greenrust2(SO4) -51.768

Romerite -57.62

Schwertmannite -73.5

Greenrust(SO3) -77.531

Copiapite -93.345

Ferricopiapite -98.436

==============================================================================
